# SHIFTING OF COGNITIVE ASSESSMENTS BETWEEN FACE-TO-FACE AND TELEPHONE ADMINISTRATION: MEASUREMENT CONSIDERATION

**DOI:** 10.1093/geroni/igae098.1349

**Published:** 2024-12-31

**Authors:** Jason Smith

**Affiliations:** Johns Hopkins University, Baltimore, Maryland, United States

## Abstract

There is limited information concerning differences in cognitive performance attributable to assessment (telephone versus in-person) method instead of cognition. We evaluated mode effects on individual cognitive items and overall cognitive score in adults aged 65-79 in the 2014 Health and Retirement Study (n=6825). We assessed mode differences in test means and reliability, whether mode modifies associations of cognition with criterion variables, and formal measurement invariance testing by mode. People assessed by telephone tended to have higher scores for memory and calculation items (0.06-0.013 SDs) and lower scores for non-memory items (-0.09 to -0.01 SD). Cognition was differentially related to IADL score depending on assessment mode. Measurement invariance testing identified the largest mode differences in memory and attention items. Differences by telephone versus face-to-face mode of administration are apparent in cognitive measurement in older adults, and most pronounced for memory and attention tests that can be easier to answer via telephone.

